# Transcriptional Mechanisms Controlling miR-375 Gene Expression in the Pancreas

**DOI:** 10.1155/2012/891216

**Published:** 2012-06-20

**Authors:** Tali Avnit-Sagi, Tal Vana, Michael D. Walker

**Affiliations:** Department of Biological Chemistry, Weizmann Institute of Science, Rehovot 76100, Israel

## Abstract

MicroRNAs (miRNAs) are a class of small non-coding RNAs that play an important role in mediating a broad and expanding range of biological activities. miR-375 is expressed selectively in the pancreas. We have previously shown that selective expression of miR-375 in pancreatic beta cells is controlled by transcriptional mechanisms operating through a TATA box-containing promoter. Expression of miR-375 has been reported in non-beta cells within the endocrine pancreas, and indeed inactivation of miR-375 leads to perturbation in cell mass and number of both alpha and beta cells. Consistent with its expression throughout the endocrine pancreas, we now show that the promoter of the miR-375 gene shows selective activity in pancreatic endocrine alpha cells, comparable to that observed in beta cells. We previously identified a novel negative regulatory element located downstream of the miR-375 gene transcription start site. By generating luciferase reporter genes, we now show that the sequence is functional also when positioned upstream of a heterologous promoter, thus proving that the repressor effect is mediated at least in part at the level of transcription. Further characterization of the transcriptional control mechanism regulating expression of miR-375 and other pancreatic miRNAs will contribute to a better understanding of pancreas development and function.

## 1. Introduction

In order to accomplish their specialized functions in the controlled secretion of metabolic hormones, pancreatic endocrine cells must strictly regulate expression of a characteristic repertoire of genes. This is mediated through a complex set of transcriptional and post-transcriptional control mechanisms [1, 2]. Recently miRNAs have been shown to play an important role in this process: thus, experiments involving conditional deletion of Dicer, an enzyme required for miRNA maturation, have revealed involvement of miRNAs in the development of both exocrine and endocrine pancreas compartments [3], as well as in mature beta cell function [4].

Although much effort has been devoted to understanding the mechanisms underlying miRNA-mediated inhibition of gene expression [5, 6], less is known concerning the mechanisms controlling expression of miRNA. This is in part due to technical limitations: many miRNA genes are embedded within exons or introns of other genes [7], complicating the analysis of transcription and processing patterns. Furthermore, identification of transcription start sites, a common first step in identification of transcriptional promoters is more challenging for genes encoding miRNAs, since the 5′ terminal sequence of the primary transcript is rapidly processed.

It has been demonstrated that the miR-375 gene is expressed selectively in cells of the endocrine pancreas [8, 9]. Indeed miR-375 is essential for endocrine pancreas function, since inactivation leads to impaired glucose homeostasis involving increased alpha cell mass and decreased beta cell mass [10]. We have examined the mechanisms underlying this selective expression of miR-375. We have characterized the promoter of the miR-375 gene, demonstrated that it shows preferential activity following transfection of beta cell lines, and have identified a number of critical cis elements within the promoter region [11]. We now report that the promoter is also selectively active in alpha cells and have characterized the role of key cis elements for promoter activity. We also have further characterized a negative regulatory element located within the transcribed region of the miR-375 gene and demonstrate that this element operates at the level of transcription.

## 2. Materials and Methods

### 2.1. Cell Culture

The following established cell lines were used in this study: HIT M2.2.2 (hamster *β* cells) [12], *β*TC1 (mouse *β* cells) [13], *α*TC1 (mouse *α* cells) [14], NIH-3T3 (mouse fibroblast cells), MIN6 (mouse *β* cells) [15], Ltk^−^ (mouse fibroblasts), and AR4-2J (rat exocrine pancreas cells) [16]. *α*TC1, *β*TC1, HIT, and NIH-3T3 cells were grown in Dulbecco's modified Eagles medium (DMEM) supplemented with 10% fetal calf serum (FCS), penicillin (200 I.U./mL), and streptomycin (100 *μ*g/mL). Ltk^−^ and AR4-2J were grown in DMEM supplemented with 10% FCS, 2 mM L-glutamine, penicillin (200 I.U./mL), and streptomycin (100 *μ*g/mL). MIN6 cells were grown on Falcon tissue culture plates in DMEM containing 10 mM D-glucose, supplemented with 15% FCS, penicillin (200 I.U./mL), and streptomycin (100 *μ*g/mL), 2 mM L-glutamine, and 72 mM beta-mercaptoethanol.

### 2.2. RNA Preparation and Quantitative PCR Reaction

RNA was extracted from *α*TC1, *β*TC1, MIN6, AR4-2J, and Ltk^−^ cells using the miRNeasy kit (QIAGEN) and was treated with Turbo-DNase (Ambion). The treated RNA (1 *μ*g) was reverse-transcribed using miScript kit (QIAGEN). Quantitative PCR reactions were carried out using 2 ng cDNA, and 200 nM oligos corresponding to miR-375, miR-106b or U6, and 7.5 *μ*L Power SYBR-Green PCR master Mix (Applied Biosystems).

miR-375 primers were 5′ TTTGTTCGTTCGGCTCGC 3′, 5′ GATTGAATCGAGCACCAGTTACG 3′; miR-106b primers were: 5′ TAAAGTGCTGACAGTGCAGAT 3′, 5′ GATTGAATCGAGCACCAGTTACG 3′; U6 primers were 5′ GATGACACGCAAATTCGTGAA 3′, 5′ GATTGAATCGAGCACCAGTTACG 3′.

Quantitative PCR reactions were performed in an AB 7300 sequence detection system. Expression of miR-375 and miR-106b was normalized to U6 RNA.

### 2.3. Plasmid Construction and Transient Transfections

Plasmid manipulations and site-directed mutagenesis were performed as previously described [11]. Transfections were carried out using the transfection reagent jetPEI (Polyplus transfection) according to the manufacturer's instructions. Forty-eight hours after transfection, cells were harvested using passive lysis buffer (Promega) and extracts were subjected to assays to determine the activity of reporter enzymes.

### 2.4. Luciferase Assays

Firefly luciferase and *Renilla* luciferase assays were carried out as follows: whole cell extracts containing 5–50 *μ*g (1–5 *μ*L) of protein were added to 100 *μ*L of either firefly luciferase assay buffer (20 mM Tricine, 0.1 mM EDTA, 1.07 mM (MgCO_3_)_4_Mg(OH)_2_∗5H_2_O, 2.67 mM MgSO_4_, 3.3 mM DTT, 270 *μ*M Coenzyme A, 470 *μ*M luciferin (Promega) and 530 *μ*M ATP, pH 7.8.) or *Renilla* luciferase assay buffer (0.1 M K_2_HPO_4_ and 0.1 M KH_2_PO_4_, pH 7.4, and 0.5 *μ*M coelenterazine (Calbiochem)). The samples were placed in a luminometer (LUMAC Biocounter M2500 or Modulus microplate, Turner Biosystems) and light output was determined over a 10-second interval. Firefly luciferase activity was normalized to the activity of* Renilla* luciferase.

## 3. Results

**Figure 1:**
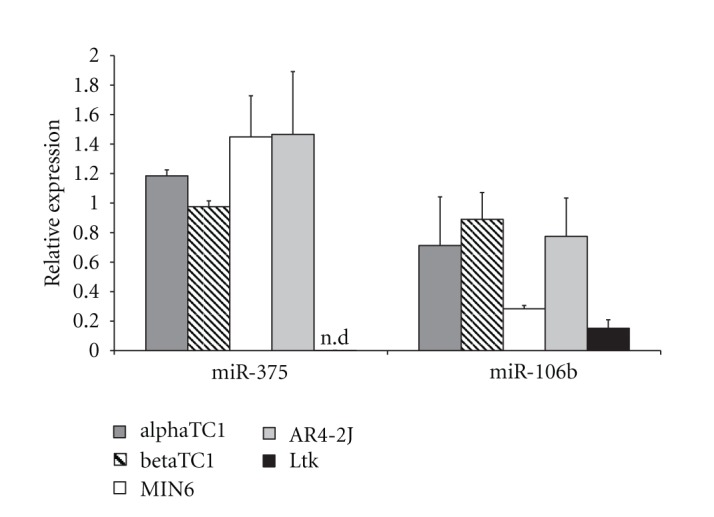
Expression levels of miR-375 and miR-106b in beta cell lines (*β*TC1 and MIN6), alpha cell line (*α*TC1), exocrine cell line (AR4-2J), and fibroblasts (Ltk^−^). Expression of the miRNAs was measured by qRT-PCR and calculated using the absolute quantification method. Results are expressed relative to U6 RNA (mean ± SEM; *n* ≥ 3), nd: not detectable.

Previous studies have revealed that miR-375 is expressed selectively in pancreatic islets [9, 10]. In order to compare expression in several pancreatic cell lines, we performed quantitative reverse transcriptase PCR (qRT-PCR) analysis on samples of RNA isolated from cell lines derived from pancreatic endocrine alpha cells (*α*TC1), pancreatic endocrine beta cells (*β*TC1 and MIN6), pancreatic exocrine cells (AR4-2J), and from non-pancreatic cells (Ltk^−^). We observed comparable levels of miR-375 in all the pancreatic cells tested, including exocrine cells: on the other hand, levels of miR-375 were at least 100-fold lower in non-pancreatic cells (Ltk^−^) (Figure 1). For comparison, we determined expression of miR-106b, which is expressed in a broad range of cell types (http://www.microrna.org/). While expression of miR-106b was similar among the pancreatic lines tested, lower yet still significant levels of expression were observed in Ltk^−^ cells. These results confirm the expression of miR-375 in pancreatic alpha cells and represent the first demonstration of expression of miR-375 in exocrine pancreas cells. Expression may not have been observed previously because high levels of RNase in exocrine pancreas tissue can make detection of RNA technically challenging.

**Figure 2:**
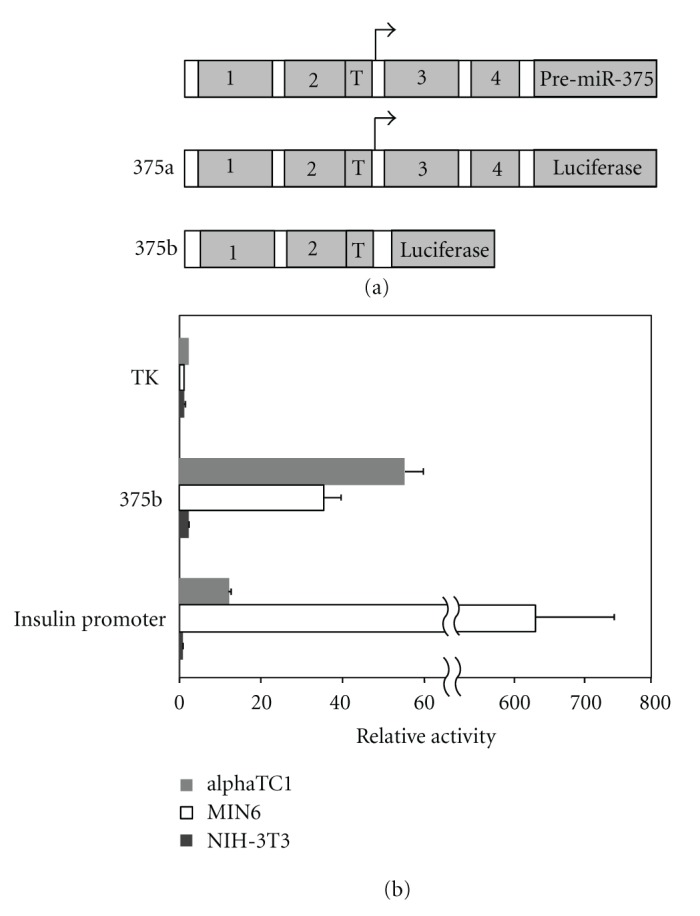
Cell specificity of the miR-375 promoter. (a) Representation of the location of conserved sequence blocks in the miR-375 gene and derived reporter gene constructs. (b) Promoter region of miR-375 (construct 375b) was ligated upstream to the firefly luciferase reporter gene in the promoter-less vector pGL3-basic. The plasmid was compared with luciferase reporter gene plasmids containing the cell-specific insulin gene promoter and the constitutive TK promoter. Promoter activity of each construct was determined following transfection into the alpha cell line *α*TC1, beta cell line MIN6, and non-pancreatic cell line NIH-3T3. Firefly luciferase activity was measured and normalized for transfection efficiency according to the activity of a cotransfected *Renilla* luciferase plasmid. Values are expressed relative to the activity of promoter-less vector pGL3-basic. Each data point represents mean ± SEM (*n* ≥ 3).

Our previous studies using transfection experiments with luciferase reporter genes revealed that the miR-375 gene promoter shows preferential activity in pancreatic beta cells lines [11]. In order to determine whether the miR-375 promoter is active also in pancreatic alpha cells, we used the cell line *α*TC1 which was generated from transgenic mice in a manner analogous to that used for the *β*TC1 cell line [14]. Indeed, we observed that the promoter shows strong activity in *α*TC1 cells, similar to that observed in beta cells, but much lower activity in the non-beta cell line NIH-3T3 (Figure 2(b)). In contrast, the TK promoter shows a similar low level of activity in all 3 cell lines tested (2.5 in *α*TC1, 0.6 in MIN6 and 1.3 in NIH-3T3, Figure 2(b)), whereas the insulin promoter shows strongly preferential activity in beta cells, as compared to alpha cells and non-pancreatic cells (Figure 2(b)). Thus the selective expression of miR-375 observed in alpha cells is at least partly attributable to transcriptional control mediated through the miR-375 promoter. The fact that the relative promoter activity is somewhat higher in *α*TC1 cells as compared to MIN6, whereas miR-375 levels are similar or slightly lower in *α*TC1 cells than in MIN6 cells, indicates that post-transcriptional control mechanisms may also play a significant role.

The data presented here showing expression of the miR-375 gene and preferential activity of the miR-375 promoter in alpha cell lines are consistent with previous results based on alpha cells from normal islets: for example, the alpha cell phenotype of mice lacking the miR-375 gene [10] and the activity of the miR-375 promoter in alpha cells of transgenic mice [11].

**Figure 3:**
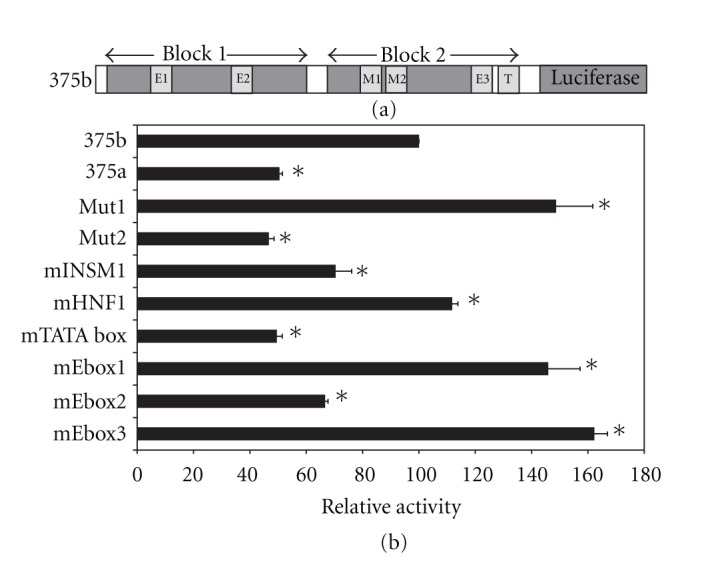
Mutational analysis of miR-375 promoter in alpha cells. (a) Representation of miR-375 gene promoter showing sequence blocks that were subjected to mutational analysis [11]. (b) Luciferase constructs containing promoter mutations were transfected into *α*TC1 cells. Luciferase activity is expressed relative to wild-type promoter activity (construct 375b). Each data point represents mean ± SEM (*n* ≥ 3; *P < 0.01, as compared to luciferase activity of construct 375b). In Mut1 (M1) the sequence TCCATGAG was mutated to TCCTCGAG, in Mut2 (M2) the sequence TATTTGCCCC was mutated to TATGTGCACC, in mE box 1 the sequence AGCCAGGTGGGT was mutated to AGCACTAGTGGT, in mE box 2 the sequence TGACATCTGGTG was mutated to TGAACGCGTGTG, in mE box 3 the sequence GGACAGGTGTG was mutated to GGGACCCGTTG, in mTATA the sequence GCAGTATAAGAG was mutated to GCAGGCGCCGAG.

**Figure 4:**
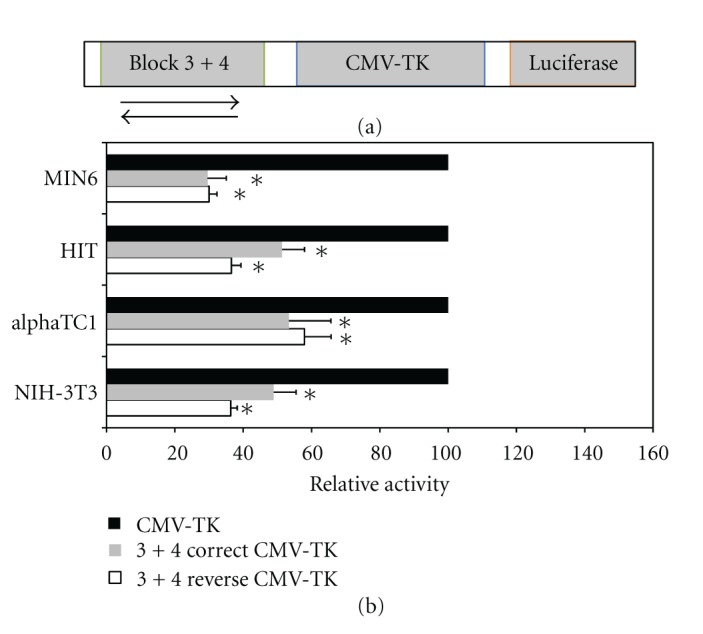
Characterization of the inhibitory effect of blocks 3 and 4. (a) Representation of plasmids containing blocks 3 and 4 inserted in both orientations upstream to the CMV-TK promoter, controlling the firefly luciferase reporter gene. (b) Luciferase activity was measured following transfection into the beta cell lines MIN6 and HIT, the alpha cell line *α*TC1, and the non-pancreatic cell line NIH-3T3. Activity is expressed relative to luciferase activity of the promoter-less vector pGL3-basic. Each data point represents mean ± SEM (*n* ≥ 3; *P < 0.05, as compared to the activity of CMV-TK promoter).

In our previous studies, we observed that a number of conserved cis elements within the promoter region were essential for full activity in transfected beta cells. These included E boxes, the TATA region, and a short region spanning the consensus binding sites for the transcription factors HNF1/INSM1 [11]. To evaluate the significance of these sequences for expression in alpha cells, we transfected luciferase plasmids containing mutated promoter sequences to *α*TC1 cells. We observed a pattern quite similar to that seen for the beta cell line HIT [11], namely, that mutations in E box 2, the TATA box, and the Mut2 regions caused a significant reduction in promoter activity (Figure 3(b)). Within the Mut2 region, mutation of the INSM1 site but not the HNF1 site led to reduced promoter activity (Figure 3(b), [11]). Thus a similar combination of transcription factors probably participates in the transcriptional control of the miR-375 promoter. Furthermore, we observed that the presence of conserved blocks 3 and 4 led to a reduction in promoter activity in alpha cells (compare constructs 375a and 375b, Figures 2(a), 3(b)). This is similar to results previously observed in beta cells, and is consistent with the notion that this region functions to inhibit transcription of the promoter. Since the sequences of blocks 3 and 4 are located downstream of the transcription start site, they could conceivably influence gene expression either at a transcriptional or post-transcriptional level. In order to distinguish these possibilities, we built constructs in which blocks 3 and 4 are located upstream of the strong CMV-TK promoter in two orientations. In such a situation, any effect of the block 3-4 region on reporter gene activity can only be mediated transcriptionally. Indeed, we observed that in alpha cells, beta cells and non-pancreatic cells, the block 3-4 region in both orientations was able to reduce significantly reporter gene expression (Figure 4).

## 4. Discussion

Since miRNAs play a central role in a broad range of biological processes including development, apoptosis, and carcinogenesis, their intracellular concentration must be tightly regulated. Indeed, expression profiles of miRNAs differ substantially among cell types [17]. In principle, this regulation may be controlled at the transcriptional or post-transcriptional level, yet the exact contribution of each of these is unknown. Furthermore, there is relatively little information available regarding the transcription of miRNA genes and the promoter elements that control the process. According to their location in the genome, miRNA genes can be classified as intragenic and intergenic. Though some intronic miRNAs have been reported to have their own promoters [18], intragenic miRNAs are generally thought to be regulated by their host gene promoter [19]. Transcription of miRNA is believed to be mediated by RNA pol II [20], though examples of involvement of Pol III have been reported [21].

We have focused on miR-375, since it shows a clear pattern of preferential expression in pancreatic cells. miR-375 was originally reported as selectively expressed in pancreatic endocrine alpha and beta cells [8, 9], and expression was subsequently reported in the gastrointestinal tract [22] and pituitary [10]. miR-375 is essential both in developing and adult pancreas: mice lacking miR-375 displayed impaired balance of alpha/beta cells, as well as defective beta cell proliferation in response to increased insulin demand [10]. Our previous results have demonstrated that selective expression in beta cells is controlled at the transcriptional level by an evolutionarily conserved promoter. We have now examined activity of the promoter in alpha cells and find that the promoter exhibits similar transcription selectivity; furthermore mutations in several key cis elements previously demonstrated to be crucial for activity in beta cells, are also important in alpha cells. This indicates that a similar spectrum of transcription factors contributes to selective expression. Based on the cis elements identified, these factors are likely to include bHLH proteins such as BETA2/NeuroD and the transcription factor INSM1. The precise role of miR-375 in alpha cells is unknown: although it may share some target genes with beta cells, loss of the miR-375 gene affects alpha and beta cells differently [10] indicating important target gene differences. The increased alpha cell mass and hyperglucagonemia observed in mice lacking miR-375 are consistent with a role for miR-375 in regulating alpha cell genes that control cell growth and proliferation, and normal glucose sensing.

An intriguing feature that we identified previously in the miR-375 gene promoter is the existence of a negative element located immediately downstream of the transcription start site within the primary transcript. We have found that blocks 3 and 4 repress promoter activity in pancreatic and non-pancreatic cells both in the context of the miR-375 gene promoter, and also when located upstream of a heterologous promoter. This clearly indicates that the repression activity of blocks 3 and 4 operates through transcriptional mechanisms and functions in several cell types. Using bioinformatic tools, we have identified binding sites for several inhibitory transcription factors within blocks 3 and 4, including Krueppel-like transcription factors, ETS1 factors and neuron-restrictive silencer factor (NRSF). Further analysis will be required to verify the transcription factors involved in this interesting repression activity, and to determine whether the block 3 and 4 regions may also be involved in post-transcriptional regulation.
